# Effect of genotype × alcoholism interaction on linkage analysis of an alcoholism-related quantitative phenotype

**DOI:** 10.1186/1471-2156-6-S1-S120

**Published:** 2005-12-30

**Authors:** Rector Arya, Thomas D Dyer, Diane M Warren, Christopher P Jenkinson, Ravindranath Duggirala, Laura Almasy

**Affiliations:** 1 Division Clinical Epidemiology, Department of Medicine, University of Texas Health Science Center at San Antonio, 7703 Floyd Curl Drive, San Antonio, TX 78229-3900 USA; 2 Department of Genetics, Southwest Foundation for Biomedical Research, P.O. Box 760549, San Antonio, TX 78245-0549 USA

## Abstract

Studies have shown that genetic and environmental factors and their interactions affect several alcoholism phenotypes. Genotype × alcoholism (G×A) interaction refers to the environmental (alcoholic and non-alcoholic) influences on the autosomal genes contributing to variation in an alcoholism-related quantitative phenotype. The purpose of this study was to examine the effects of G×A interaction on the detection of linkage for alcoholism-related phenotypes.

We used phenotypic and genotypic data from the Collaborative Study on the Genetics of Alcoholism relating to 1,388 subjects as part of Genetic Analysis Workshop 14 problem 1. We analyzed the MXDRNK phenotype to detect G×A interaction using SOLAR. Upon detecting significant interaction, we conducted variance-component linkage analyses using microsatellite marker data. For maximum number of drinks per a 24 hour period, the highest LODs were observed on chromosomes 1, 4, and 13 without G×A interaction. Interaction analysis yielded four regions on chromosomes 1, 4, 13, and 15. On chromosome 4, a maximum LOD of 1.5 at the same location as the initial analysis was obtained after incorporating G×A interaction effects. However, after correcting for extra parameters, the LOD score was reduced to a corrected LOD of 1.1, which is similar to the LOD observed in the non-interaction analysis. Thus, we see little differences in LOD scores, while some linkage regions showed large differences in the magnitudes of estimated quantitative trait loci heritabilities between the alcoholic and non-alcoholic groups. These potential hints of differences in genetic effect may influence future analyses of variants under these linkage peaks.

## Background

Family, twin, and adoption studies have indicated that genetic and environmental factors and their interactions contribute to the development of alcoholism [1-3]. Several studies have demonstrated the importance of considering environment-specific major gene effects on different phenotypes. Genotype × alcoholism (G×A) interaction refers to the environmental (alcoholic and non-alcoholic) influences on the autosomal genes contributing to variation in an alcoholism-related quantitative phenotype. Alcoholic environment refers to chronic alcohol ingestion. This may interact with gene expression in a number of different ways; affected individuals carry a different spectrum of genetic variants; or developmental differences between individuals, who are or are not at risk for alcoholism, affect gene expression; or ingestion of alcohol affects gene expression. Although it is well documented that alcoholism-related traits have strong genetic determinants, few susceptibility genes influencing these complex disease phenotypes have been identified. Because alcoholism is a complex phenotype influenced by several genes with small effects, it is difficult to detect such genes. Hence it may be helpful to examine potentially simpler endophenotypes related to disease risk [4]. On the other hand, it may be easier to detect such susceptibility genes if they have major effect on related quantitative phenotypes [5-7]. In addition, gene × environment (G×E) interaction has been detected in quantitative genetic analyses of a variety of traits such as serum lipid concentrations and event-related evoked potentials (ERPs) [8,9]. Furthermore, G×E interactions (e.g., genotype × age, genotype × sex, and genotype × diet) in a given quantitative trait (e.g., body composition and ERP phenotypes) under the assumption of polygenic inheritance has been considered an important component in modeling environment-specific effects for polygenic variance components and major genes [9-12]. Therefore, in this study, we examined the effects of G×A interactions on the linkage analysis of a quantitative phenotype from Collaborative Study on the Genetics of Alcoholism (COGA) data, maximum number of drinks per a 24 hour period (MXDRNK), which is a correlate of alcoholism and is expected to reflect individual's ability to metabolize alcohol as well as the effect of social environment. By using alcoholism as an environment in G×E analyses of MXDRNK (i.e., we are referring to internal/within individual environment but not family environment), we are essentially allowing for the possibility that the magnitude or source of genetic effects on variation in alcohol consumption may differ in alcoholics and non-alcoholics.

## Subjects and Methods

In this study, the Genetic Analysis Workshop 14 (GAW14) COGA data (Problem 1) consisting of 1,388 family members, have been analyzed. Prior to the analysis we recoded the affection status based on the definition of alcoholism according to COGA as well as DSM-IV criteria in two ways: diagnoses 1 and 2 correspond to COGA and DSM-IV and that a includes individuals with some symptoms as unaffected (diagnoses COGA-Aldxla and DSM-IV-Aldx2a), whereas b considers them unknowns (diagnoses COGA-Aldx1b and DSM-IV-Aldx2b). In the analysis of the GAW14 COGA data, we used a maximum likelihood variance components approach for the study of G×E interaction using related individuals in different environments [10]. To minimize the problem of non-normality, MXDRNK values were log transformed. In this interaction model, two additional parameters are modeled: a) environment-specific genetic variances, and b) a genetic correlation between groups of individuals living in different environments. A significant G×E interaction is indicated by significantly different magnitudes of genetic variances for individuals living in different exposure groups (alcoholics vs. non-alcoholics), and/or a genetic correlation (ρ_G_) is less than 1 between exposure groups. In an interaction model, assuming the probability of an individual having a specific polygenotype is independent of environment, the expected additive genetic covariance between a pair of alcoholics is COV_(alc,alc) _= 2Φ σ^2^_Galc _or the covariance between a pair of non-alcoholics would be COV_(noalc,noalc) _= 2Φ σ^2^_Gnalc_, i.e., 2Φ times the appropriate genetic variance.

The covariance between an alcoholic individual and a nonalcoholic one is modeled as:

COV_(alc,nalc) _= 2Φ σ_Galc _σ_Gnalc _ρ_G_,

where alc and nalc denote alcoholics and non-alcoholics, respectively, Φ is the coefficient of kinship between the two individuals, ρ_G _is the additive genetic correlation between the expression of the phenotype in the two environments, and σ_Galc _and σ_Gnalc _are the additive genetic standard deviations of alcoholics and nonalcoholics, respectively. We first screened for the presence of G×A interaction in several quantitative phenotypes, including MXDRNK, using a quantitative genetic method. After detecting significant G×A interaction for MXDRNK phenotype, we performed variance-component linkage analyses with a customized model to include diagnosis-specific quantitative trait loci (QTL) effects and using microsatellite marker multipoint identity by descent (MIBD) matrices estimated using LOKI. The customized variance component linkage model may be defined as

COV_(alc,nalc) _= Π σ_Qalc _σ_Qnalc _+ 2Φ σ_Galc _σ_Gnalc _ρ_G_.

This model has an additional QTL variance as compared with the standard linkage model. Corrected LOD (LOD_c_) scores assume that σ_Qalc _σ_Qnalc _are independent under the null, producing a test statistic distribution of ¼χ^2^_2_, ½χ^2^_1_, ¼ point mass at 0. This assumption may be overly conservative. These analytical techniques were implemented using the computer program SOLAR [13].

## Results

Results of G×A interaction analyses for several quantitative phenotypes according to two diagnostic criteria with a modified coding for affection status are shown in Table 1. The particular subset of electrophysiological phenotypes analyzed was chosen on the basis of significant or suggestive genome-wide linkage results [see [14]]. Of the examined phenotypes, LNMXDRNK showed significant differences in magnitude of genetic effects (gsd) between alcoholics and non-alcoholics and the correlation in genetic effects (ρ_G _< 1), suggesting different genetic effects in the two environments. It is necessary to replicate this finding in the entire COGA sample to make sure that it is not a false positive. But if we correct for multiple testing, i.e., by multiplying p-values by 28 (7 traits × 2 diagnoses × 2 parameters tested), MXDRNK is still significant (*p *< 0.028).

**Figure 1:**
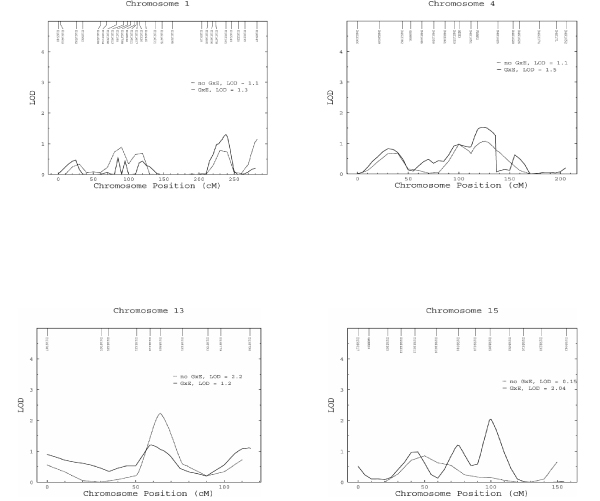
Linkage of MXDRNK phenotype to genetic locations on chromosomes 1, 4, 13, and 15 with and without G×A interaction effects in COGA data.

Descriptive statistics for the MXDRNK phenotype based on diagnostic criteria and affection status are reported in Table 2. The MXDRNK phenotype exhibited moderate but significant heritability (h^2 ^± SE, LNMXDRNK = 0.18 ± 0.05, *p *< 0.0001) after adjusting for age, sex, and smoking influences. For MXDRNK, chromosomal regions with LODs > 1 obtained in both linkage analyses (non-G×A and with G×A) are presented in Table 3 and Figure 1. For MXDRNK, the highest LODs were observed on chromosomes 13 (2.2 at 64 cM), 4 (1.1 at 126 cM), and 1 (1.1 at 282 cM) in non-G×A interaction analysis. Interaction analysis yielded a region on chromosome 1 with a LOD of 1.3 (corrected LOD (LOD_c_) = 0.9 at 238 cM) near the marker D1S2141, which is located 44 cM centromeric to a region obtained initially in the analysis without G×A interaction. The chromosomal region near marker D4S1651 on chromosome 4 showed improved evidence for linkage (LOD = 1.5, LOD_c _= 1.1) to the MXDRNK phenotype incorporating G×A interaction (Aldx1b diagnosis) near marker D4S1651 (126 cM). On chromosome 13, the initial analysis yielded a max LOD of 2.24 at 64 cM near marker D13S800, while the interaction analysis showed a reduced LOD of 1.21 (LOD_c _= 0.8) at 59 cM in almost the same region between markers D13S318 and D13S800. Also, an additional region with a max LOD of 2.04 (LOD_c _= 1.6) at 100 cM was obtained near marker D15S205 in the G×A interaction analysis.

## Discussion

Environment may influence the variation in the expression of genes influencing a variety of phenotypes including alcohol-related phenotypes. Genotype × alcoholism interaction was explored for a variety of quantitative phenotypes in the COGA dataset but was detected only for the MXDRNKs phenotype. Results of G×A analyses were consistent across the COGA and DSM-IV alcoholism diagnoses, but were affected by the categorization of individuals who were unaffected with some symptoms. Results were generally stronger when these individuals were categorized as unaffected, rather than unknown. However, this result may be strongly influenced by sample size considerations as the addition of the "unaffected with some symptoms" more than doubled the size of the nonalcoholic group.

Our analysis also shows that accounting for G×E interaction may increase the linkage signal. For MXDRNK, interaction analysis failed to show evidence at the implicated region on chromosome 1 in the non-G×A analysis but yielded a slightly increased linkage signal at a different location (238 cM), which corresponds to the previously reported linkage with factor 2, a factor analysis-derived trait defined by harm avoidance, novelty seeking, and age of onset of drinking [15]. On chromosome 4, although the increase in LOD score is only slightly higher than the LOD obtained in the linkage model without interaction, the observed environment-specific QTL effects are interesting and are consistent with the previous observation that inclusion of unaffected individuals is crucial to detection of linkage to this chromosome 4 region. Moreover, chromosome 4 QTL appears to influence unaffecteds only and this observation is also consistent with COGA findings Furthermore, this region is important because this QTL has an impact on drinking behavior in non-alcoholics and it is consistent with that identified in other studies in the literature [5,16]. Interestingly, interaction analysis has also yielded a new region on chromosome 15 (max LOD = 2.04, LOD_c _= 1.6, 100 cM), which is only 25 cM away from a linkage with factor 2 [15] and QTL effect is stronger in affecteds. On the other hand, no signal was observed at the corresponding region in non-G×A analysis. In contrast, the evidence for linkage has been reduced on chromosome 13 in G×A interaction analysis and the QTL effect appears to be stronger in unaffecteds.

## Conclusion

In conclusion, genotype × alcoholism interaction analysis yielded interesting results. Drinking behavior appears to be influenced by environment-specific genes in both alcoholics and non-alcoholics. The implicated regions on chromosomes 1, 4, and 15 are consistent with previously reported linkage findings. These results indicate that further analyses may benefit from considering the possibility of differing genetic effects in alcoholics and non-alcoholics, for example by stratifying analysis on alcoholism diagnosis.

## Abbreviations

COGA: Collaborative Study on the Genetics of Alcoholism

ERP: Event-related evoked potentials

G×A: Genotype × alcoholism

G×E: Genotype × environment

GAW14: Genetic Analysis Workshop 14

MIBD: Multipoint identity by descent

MXDRNK: Maximum number of drinks per a 24-hour period

QTL: Quantitative trait loci

## Authors' contributions

RA contributed to the study design, performed the genetic analyses, interpreted the results, and prepared the manuscript. LA has contributed the study design, methodology, and interpretation of results. TDD provided MIBDs. DMW ran initial linkage screen based on which traits were selected for this study. RD and CPJ participated in the interpretation of results.

## Figures and Tables

**Table 1:** *p*-Values for the two tests of G×A interaction (gsd_alc _= gsd_nalc _and ρ_G _= 1) for several quantitative phenotypes.

		*p*-Values			
		
		COGA		DSM-IV	
			
Trait	G×A interaction^a^	Aldx1a	Aldx1b	Aldx2a	Aldx2b
lnMXDRNK	gsd_alc _= gsd_nalc_	0.359	0.001	0.242	0.018
	ρ_G _= 1	0.118	0.006	0.006	0.071
cigpky	gsd_alc _= gsd_nalc_	0.452	0.639	0.728	0.329
	ρ_G _= 1	0.067	0.075	0.004	0.038
ecb211	gsd_alc _= gsd_nalc_	0.145	0.730	0.246	0.397
	ρ_G _= 1	1.000	1.000	0.450	0.496
ttth1	gsd_alc _= gsd_nalc_	0.729	0.804	0.772	0.853
	ρ_G _= 1	0.352	1.000	0.211	1.000
ttth3	gsd_alc _= gsd_nalc_	0.453	0.510	0.425	0.971
	ρ_G _= 1	0.362	0.348	0.227	0.227
ttdt3	gsd_alc _= gsd_nalc_	0.829	0.793	0.814	0.695
	ρ_G _= 1	0.135	0.493	0.200	0.081
ntth4	gsd_alc _= gsd_nalc_	0.172	0.702	0.236	0.716
	ρ_G _= 1	1.000	1.000	1.000	1.000

^a ^gsd_alc _= gsd_nalc _= difference in magnitude; ρ_G _(correlation in genetic effects) = 1

**Table 2:** Descriptive statistics of MXDRNK phenotype according to diagnostic criteria and affection status

	MXDRNK				
	
Diagnostic Criteria	Affection status^a^	Mean	Variance	Range	*n*
ALDX1	1	4.25	11.41	1.0–30.0	285
	2	0.00	0.00	0.0	29
	3	12.87	95.32	4.0–72.0	431
	5	25.41	186.94	5.0–72.0	621
ALDX2	1	4.25	11.41	1.0–30.0	285
	2	0.00	0.00	0.00	29
	3	14.42	106.89	4.0–68.0	531
	5	26.24	198.99	5.0–72.0	521

^a^1, pure unaffected; 2, never drank; 3, unaffected with some symptoms; 5, affected

**Table 3:** Chromosomal regions linked to MXDRNK phenotype in COGA data

Chr	Marker region	cM	(non-G×A)	(with G×A)	Corrected LOD	qsd_alc _(aff)^a^	qsd_nalc _(unaff)^b^
1	D1S547	282	1.14	0.20	0.05	1.43	1.52
1	D1S2141	238	0.73	1.29	0.92	2.11	2.00
4	D4S1651	126	1.07	1.52	1.12	0.00	3.29
13	D13S800	64	2.24	1.06	0.71	1.22	2.63
13	D13S318–D13S800	59	1.6	1.21	0.84	0.79	2.85
15	D15S205	100	0.15	2.04	1.6	2.75	0.46

^a ^aff, affected

^b ^unaff, unaffected
